# Exploring the Dual Interaction of Natural Rhamnolipids with Plant and Fungal Biomimetic Plasma Membranes through Biophysical Studies

**DOI:** 10.3390/ijms20051009

**Published:** 2019-02-26

**Authors:** Noadya Monnier, Aurélien L. Furlan, Sébastien Buchoux, Magali Deleu, Manuel Dauchez, Sonia Rippa, Catherine Sarazin

**Affiliations:** 1 Unité de Génie Enzymatique et Cellulaire, CNRS UMR 7025, Université de Picardie Jules Verne (UPJV), 80039 Amiens, France; noadya@gmail.com (N.M.); furlanaurelien@gmail.com (A.L.F.); sebastien.buchoux@u-picardie.fr (S.B.); 2 Unité de Génie Enzymatique et Cellulaire, CNRS UMR 7025, Sorbonne Universités, Université de Technologie de Compiègne, 60200 Compiègne, France; sonia.rippa@utc.fr; 3 Laboratoire de Biophysique Moléculaire aux Interfaces, Gembloux Agro-Bio Tech, B5030 Gembloux, Belgium; magali.deleu@uliege.be; 4 Matrice Extracellulaire et Dynamique Cellulaire, UMR CNRS 7369, Chaire MAgICS, Université de Reims Champagne-Ardenne (URCA), 51687 Reims, France; manuel.dauchez@univ-reims.fr

**Keywords:** rhamnolipid, membrane insertion and dynamic, biomimetic lipid membrane, plant plasma membrane, fungi plasma membrane, membrane sterol, phytosterols, ergosterol

## Abstract

Rhamnolipids (RLs) are potential biocontrol agents for crop culture protection. Their mode of action has been proposed as dual, combining plant protection activation and antifungal activities. The present work focuses on the interaction of natural RLs with plant and fungi membrane models at the molecular scale. Representative models were constructed and the interaction with RLs was studied by Fourier transform infrared (FTIR) and deuterium nuclear magnetic resonance (^2^H NMR) spectroscopic measurements. Molecular dynamic (MD) simulations were performed to investigate RL insertion in lipid bilayers. Our results showed that the RLs fit into the membrane models and were located near the lipid phosphate group of the phospholipid bilayers, nearby phospholipid glycerol backbones. The results obtained with plant plasma membrane models suggest that the insertion of RLs inside the lipid bilayer did not significantly affect lipid dynamics. Oppositely, a clear fluidity increase of fungi membrane models was observed. This effect was related to the presence and the specific structure of ergosterol. The nature of the phytosterols could also influence the RL effect on plant plasma membrane destabilization. Subtle changes in lipid dynamics could then be linked with plant defense induction and the more drastic effects associated with fungal membrane destabilization.

## 1. Introduction

**Figure 1:**
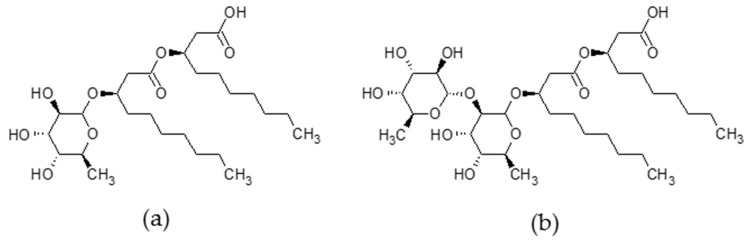
Chemical structure of main *Pseudomonas aeruginosa* rhamnolipids (RLs). (**a**) Main mono-RL: α-l-rhamnopyranosyl-β-hydroxydecanoyl-β-hydroxy-decanoate; (**b**) Main di-RL: 2-*O*-α-l-rhamnopyranosyl-α-l-rhamnopyranosyl-β-hydroxydecanoyl-β-hydroxydecanoate.

Rhamnolipids (RLs) are secondary metabolites naturally produced by bacteria of the genera *Pseudomonas* and *Burkholderia* with biosurfactant properties. Rhamnolipids are produced as a mix of compounds with one or two rhamnose residues (mono- or di-RLs) forming a polar head and linked through a beta-glycosylic bond to one or two 3-hydroxy fatty acids. *Pseudomonas aeruginosa* RLs, which are the most studied RLs, are produced as a mixture of mainly α-l-rhamnopyranosyl-β-hydroxydecanoyl-β-hydroxy-decanoate and 2-*O*-α-l-rhamnopyranosyl-α-l-rhamnopyranosyl-β-hydroxydecanoyl-β-hydroxydecanoate [1], (Figure 1). Those compounds were first studied for their potential in bioremediation and removal of hydrophobic organic pollutants and heavy metals [2,3]. As RLs’ amphiphilic properties are the key to their potential, the aggregation behavior of those biosurfactants have been extensively studied [4,5,6]. Those properties of RLs are used in detergents, food, and cosmetic industries as those compounds have a low toxicity and weak ecological impact [2,3]. Besides, their supposed antitumor activity, their role in the stimulation of innate immunity in animals, their large range antimicrobial activities, and their ability to trigger defense responses in plants greatly reinforce RLs’ potential uses in areas such as biomedicine, therapeutics, and agriculture [7,8].

The description of RLs’ biological properties has strengthened the need to describe RL interaction with cell membranes, especially with membrane lipids. Rhamnolipid interaction with lipids has been mostly investigated through the study of biomimetic membranes composed of phosphatidylcholines (PCs), the main phospholipid in many living cells [9]. Indeed, biomimetic membrane models are precious tools to facilitate the understanding of highly complex biological membrane behaviors [9]. Mono- and di-RL ability to form supramolecular complexes with saturated 1,2-dipalmitoyl-sn-glycero-3-phosphocholine (DPPC) has been shown by mass spectroscopy [10]. For di-RLs this affinity has been demonstrated to be entropy driven by isothermal titration calorimetry on liposomes of the mono-unsaturated phospholipid 1-palmitoyl-2-oleoyl-glycero-3-phosphocholine (POPC). Interestingly, the interaction of di-RLs with those liposomes are less favorable in the presence of cholesterol [11]. This interaction has been shown to allow di-RLs to induce leakages in POPC vesicles at ratios higher than one RL for one lipid, this effect being reduced in the presence of cholesterol [12]. Studies realized on saturated phospholipids, with smaller RL/lipid ratios (ranging from 1 RL for 5 to 100 lipids), have shown that di-RLs modify the gel-to-liquid-crystalline transition of 1,2-dimyristoyl-sn-glycero-3-phosphocholine (DMPC) [13]. On DPPC, both mono- and di-RLs modify polar head absorbance in Fourier transform infrared spectroscopy (FTIR) [14,15]. It can be noted that the study of liposomes composed of phospholipid and RL mixtures opened interesting possibilities in cell delivery for both cosmetic and pharmaceutics [13,16,17,18]. Globally, those studies supported the idea of a potential interaction between RLs and membrane lipids in vivo [13,16,17,18]. Still most studies focus on separated mono- or di-RLs while RLs are naturally produced as a mix [1]. Studies of RL’s mixed effects on unsaturated lipids and complex membrane models are still lacking.

The data gathered on lipid models inform on RL interaction with living cells. Studies on phosphatidylethanolamine (PE) models used to mimic the inner membranes of bacteria have shown that mono- and di-RL molecular shapes have opposite effects on those membranes [11,19,20]. Indeed, mono-RLs promote hexagonal H_II_ phase formation while di-RL presence can impede it [19,20]. Effects on membrane lipids could be associated in vivo with the inhibition of bacterial growth induced by RLs as well on both Gram-negative and Gram-positive bacteria [7]. Rhamnolipid interaction with lipids is also described as being responsible for the RL lytic activity on zoospores of the fungus *Phytophtora capsici*, as an example, through a detergent-like mechanism [21,22]. In addition, RLs also cause mycelium growth inhibition and conidia germination delays on *Botrytis cinerea* [23,24]. To our knowledge, no experiment has been conducted to date on filamentous fungi membrane models. Thus, it appears necessary to understand how RLs interact with fungi plasma membrane lipids and to study their mode of action on spore germination and mycelium growth inhibition.

Rhamnolipids are also known for their ability to trigger defense responses in plants. This property was first described on grapevines and later on the model Brassicaceae *Arabidopsis thaliana*, on cherry-tomato fruit and on the annual crop rapeseed [23,24,25,26]. Contrary to the observations on micro-organisms, RLs appear to have a positive influence on plant organisms, leading to molecular defense responses and physical protection against various pathogens. The perception mode of RLs by plant cells is not understood yet. Two hypothesis have been proposed by Varnier et al. [23]. The first assumption refers to an hypothetic specific transmembrane proteic receptor with a leucine-rich repeat extracellular domain, in the same way as for other plant molecular defense elicitors [27]. This kind of receptor driven perception is similarly supposed to be responsible for immunostimulation of human monocyte cells by specific forms of RLs [28]. In the second assumption, a perception of RLs through direct interactions with membrane lipids is suggested to trigger a signal activating the plant defense pathways. This last hypothesis has been proposed for other amphiphilic compounds like the microbial pore-former peptides harpin HrpZ and alamethicin [29,30,31,32], or the lipopeptide surfactin [33]. The ability of synthetic glycolipids containing rhamnose to trigger defense responses in plant and to interact with membrane models [34,35] reinforces this hypothesis.

The present work is based on the study of RL interactions with plant and fungi plasma membrane models taking into account the presence of unsaturation, charges, and different lipid families. This molecular scale study combines FTIR, nuclear magnetic resonance (NMR) and molecular dynamics (MDs) as complementary tools to describe exogenous molecule insertion and localization in model membranes and their influence on liposome dynamics [9,34,36]. Mono- or di-RLs and a natural mono-/di-RL mix (4/6) were used. In order to stay as close as possible to biological reality, only small amounts of RLs (around 1 RL for 20 or 50 lipids) were used in biomimetic models. Indeed, the access to membrane lipids in vivo is restricted by the cell wall in both plant and fungal cells, RL/lipid ratios are thus expected to be quite low in vivo. The same range of ratios were used to study the interaction between lipid membrane models and surfactin [36,37,38,39], which is also a small amphiphilic molecule known to have plant eliciting effect and direct antimicrobial properties [33,40]. Unless otherwise specified all experiments were conducted at pH 7.5, which correspond to deprotonated and negatively charged RLs accordingly to their p*K*a value [41]. This molecular form avoids RLs’ insolubility in water and eases their dispersion in aqueous media [42,43] favoring RL interaction with biological membranes. Moreover, it is a convenient pH for liposome formation [36,38,44]. The results strengthen the comprehension of RL interaction with plant and fungi membrane lipids, showing the favorable insertion of RLs in unsaturated PC bilayers with localization around glycerol backbone. Besides, contrary to our observations on different plant biomimetic models, RLs clearly induce an increase of membrane fluidity on a fungus membrane model. This effect is dependent on sterol nature and content and could be one of the molecular causes of the different biological activities of RLs on plants and fungi.

## 2. Results and Discussion

### 2.1. Localization of RLs in Simplified Phospholipidic Biomimetic Plant Membranes

In a first approach, the interaction of purified mono-and di-RLs was studied experimentally by FTIR to characterize interaction with di-unsaturated PC of each RL form. 1-Palmitoyl-2-linoleoyl-sn-glycero-3-phosphocholine (PLPC) was thus considered as it represents 32% of PC in the plasma membrane model plant *A. thaliana* [45]. Fourier transform infrared spectroscopy, a widely-used method for studying bioactive molecules’ interaction with phospholipidic biomimetic membranes was used to compare PLPC liposomes with and without mono- or di-RLs. The infrared (IR) spectra allows to consider the modification induced by exogenous molecules on the hydrophobic part of PC through absorbance measurements of alkyl chains (3000–2800 cm^−1^), and on the hydrophilic part through absorbance measurements of C=O bonds (1800–1400 cm^−1^) and phosphate groups (1300–1150 cm^−1^) [9].

**Figure 2:**
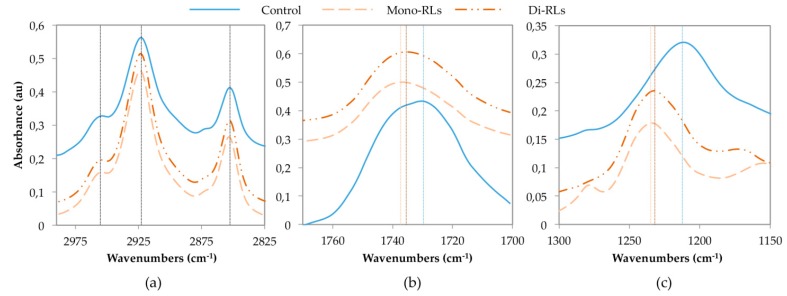
Study of rhamnolipid (RL) insertion into phosphatidylcholines (PC) bilayers by Fourier transform infrared (FTIR) at an RL/lipid ratio of 1:19. (**a**) Effect of mono- and di-RLs on absorption bands of 1-Palmitoyl-2-linoleoyl-sn-glycero-3-phosphocholine (PLPC) liposomes acyl chains; (**b**) effect on C=O bonds; and (**c**) effect on P=O bonds.

Here, maximum absorbance corresponding to PLPC terminal CH_3_ groups, at 2954 cm^−1^, and asymmetric and symmetric vibrations of CH_2_ groups at 2923 and 2852 cm^−1^, were unaffected by RLs (Figure 2a). Conversely, C=O and P=O maximum absorbance were shifted to higher wavenumbers by both mono- or di-RLs. For C=O bonds, the PLPC observed absorbance maximum was at 1729 cm^−1^, and differences of 9 and 6 cm^−1^ were respectively obtained for mono- and di-RLs (Figure 2b). Shifts of P=O bond absorption maximum were more important. The band at 1211 cm^−1^ for PLPC were displaced at 23 and 21 cm^−1^ for mono- and di-RLs, respectively (Figure 2c). The absorbance shift observed for P=O bonds clearly indicates a less hydrogen-bonded state of the phosphate group in the presence of RLs that can be related to a lesser degree of hydration of the phosphate group [46]. This effect can be due to a direct interaction between the PLPC phosphate groups and the polar heads of RLs and/or to an indirect influence of the presence of RLs on the arrangement of polar headgroups of the phospholipids. Furthermore, results obtained for mono- and di-RLs were similar, suggesting analogous interactions of the two molecules with this model. Even if slightly higher shifts were obtained with mono-RLs, those differences were smaller than the spectrometer resolution (4 cm^−1^). PLPC absorbance spectra was similar to previous results confirming data reliability [34].

These results suggest that RLs are located near the phosphate group. Moreover, as alkyl chain absorbance was unchanged after RL addition they did not affect PLPC alkyl chain C–H bond stretching. It suggests that each RL was not deeply inserted in the hydrophobic part of model membranes and located around phospholipid P=O bonds. Rhamnolipid presence near lipid polar heads seemed to be confirmed by the observed shift of C=O bond absorbance, although this difference could also be very partially associated to RL C=O bonds. This is consistent with previously reported results obtained by FTIR on (DPPC) models containing 7 to 20% of RLs [14,15]. Even at those high ratios, the wavenumber shift on acyl chain maximum absorption was under 2 cm^−1^ which confirms that natural RLs do not strongly perturb the hydrophobic part of the membrane. On this saturated PC model, a significant shift of lipid C=O bonds maximum absorbance was also observed, which is consistent with a greater effect on lipid polar heads. The comparison of these results on PLPC to data from literature on DPPC models [14,15] suggests that RLs have the same behavior with saturated and unsaturated lipids of similar chain length.

**Figure 3:**
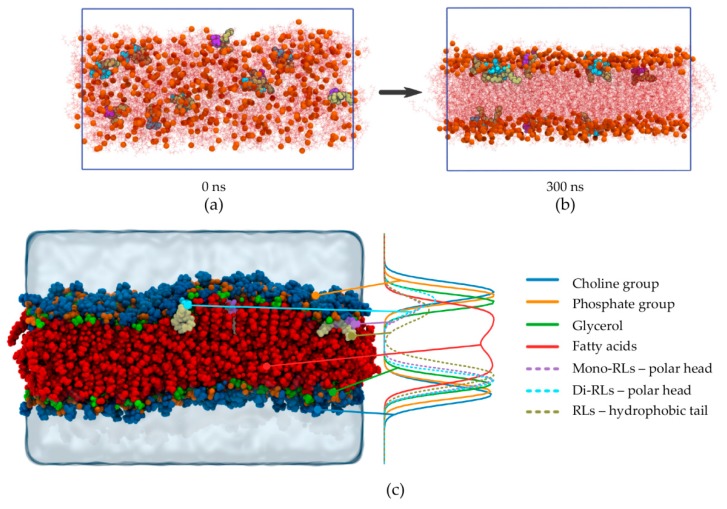
Study of RL insertion into PC bilayers by Molecular dynamic (MD) simulation of RLs (4 mono-RLs, 6 di-RLs) co-solubilized with 1-palmitoyl-2-oleoyl-glycero-3-phosphocholine (POPC)/PLPC (256 POPC, 256 PLPC). (**a**) Snapshot extracted from the trajectory beginning (0 ns); (**b**) snapshot extracted from the trajectory end (300 ns); (**c**) final snapshot and corresponding density profile. In (**a**–**c**), RLs are depicted using Van der Waals spheres and colored as follows: tan for lipid moiety, purple and cyan for mono-RL and di-RL sugar moiety, respectively. In (**a**,**b**), lipids are depicted as red lines for clarity with the exception of the phosphorus atom which is represented by an orange Van der Waals sphere. In (**c**), lipids are depicted as Van der Waals spheres and colored as follows: red for lipid chains, green for glycerol, orange for phosphate, and blue for choline. Limits of the MD box are shown by the blue line in (**a**,**b**) and by the extent of the water molecules (represented by the transparent blue volume) in (**c**).

In a second approach, MD simulations were performed to provide an atomistic description of phenomena and to enrich experimental data, taking into account the experimental behavior of each form of RL in FTIR experiment. Then, RL behavior was studied as a mix as naturally produced by *P. aeruginosa*, on a (POPC)/PLPC model, POPC being the most widely unsaturated PC used in biophysical studies [47,48,49]. An RL/lipid ratio of 1:50 was used. The simulating system was built completely randomly. This random starting configuration is shown in Figure 3a, where it can clearly be seen that RLs (4 mono-RLs and 6-diRLs) and lipids were completely mixed when the simulation began. It can be noted that all RLs were deprotonated and negatively charged accordingly to the pH used in previous experiments, which was above the RL p*K*a value [41].

During the simulation, phospholipids self-assemble to form a lipid bilayer, and as it can be seen in Figure 3b (*t* = 300 ns), all RLs end up within the bilayer and are located just below the lipid glycerol. It is important to note that no RL remains in the aqueous compartment and that RLs do not self-aggregate. In order to give a clearer indication of RL location, a density profile was realized by averaging the last 100 ns of the simulation (the equilibrium actually reached around 130 ns of simulation). As illustrated by the density profile presented in Figure 3c, sugar moieties of both mono-RLs and di-RLs (purple- and cyan-dashed lines, respectively) are located around phosphate and glycerol positions (orange- and green-solid lines, respectively) whereas RL hydrophobic chains (tan-dashed line) are located below the glycerol and closer to the lipid chains (red-solid line). The upper part of the final snapshot in Figure 3c can be interpreted in a misleading way because of the wave made by the membrane which leads in the density profile to a slight difference between the two RL forms. However, in the lower part of the density profile (Figure 3c) and in Figure 3b at 300 ns, no difference can be made in the localization of the two RL forms.

Thus, the MD simulation shows that RLs are located near the lipid phosphate group in a simple POPC/PLPC model which is consistent with experimental FTIR results obtained with a simpler PLPC model. The MD simulation allowed a more precise localization of both forms of RLs nearby the phospholipid glycerol backbone. As shown by identical position of mono- and di-RL hydrophobic tails, similar behaviors were observed for both RL forms in FTIR and molecular dynamics experiments. Taken as a whole, those results confirm a similar interaction of these two glycolipid forms with PC. The good agreement of data obtained with the two methods strongly supports the proposed position of RLs interacting with phospholipid bilayers.

### 2.2. Impact of RL Insertion on Plant Phospholipidic Plasma Membrane Model: Dynamic Insight

The thermodynamically favored position of RLs into RL containing PC bilayers appears to be at the level of phospholipid glycerol backbone. It is then important to study how RLs insert into biological membrane to reach this equilibrium.

#### 2.2.1. Kinematics of RL Insertion into Lipid Bilayers

**Figure 4:**
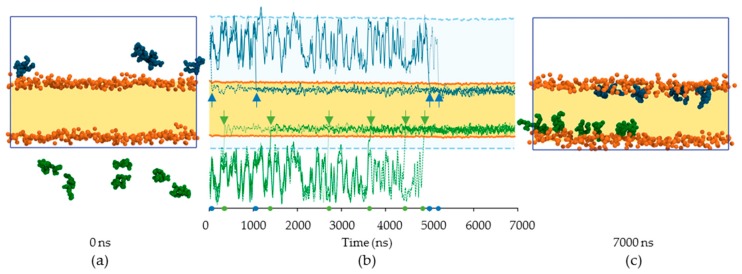
MD simulation of RL addition (4 mono-RLs, 6 di-RLs) to a pre-equilibrated POPC/PLPC bilayer (256 POPC, 256 PLPC). (**a**) Snapshot at the beginning of the simulation (0 ns). RLs are represented using Van der Waals spheres; RLs located above the lipid bilayer (orange Van der Waals spheres for phosphorus and yellow to highlight the hydrophobic core) are colored in blue and the ones below the membrane are in green (no distinction between mono-RL and di-RL is made). The MD box limits are represented by the dark-blue solid line; (**b**) evolution of each RL position along the trajectory. The positions of the lower RLs are shown by the green lines and the positions of the upper ones are shown by the blue lines (in both cases, different line styles are used for clarity). The membrane is depicted in yellow surrounded by two thick orange lines which correspond to the average positions of the lower and upper phosphorus atoms. The MD box is highlighted by the light-blue background and the two thick dashed light-blue lines which represent its extent. Each RL insertion in the membrane is indicated by an arrow and a spot on the timeline; (**c**) snapshot at the end of the simulation (7000 ns). Representations are identical to (**a**).

To assess if RLs spontaneously insert into a lipid bilayer, an MD simulation was realized with RLs added to a pre-formed lipid membrane (POPC/PLPC, 1/1). As previously, a mono-/di-RL mix was considered at a RL/lipid ratio of 1:50. The initial step is presented in Figure 4a with the lipid bilayer position (in yellow) delimited by the phosphorus atoms (orange beads) and the RLs (4 blue above the membrane, 6 green below) in water. The whole trajectory is provided as a video file (Supplementary Materials Video S1). The summary of RL insertion inside the lipid bilayer is shown in Figure 4b, and the position of the center of mass for each RL is shown (upper RLs indicated by blue lines, and lower RLs by green lines). The positions of RLs tend to fluctuate as long as they stay in water. As soon as a given RL has entered the lipid bilayer, it stays there and never exits the membrane. By the end of the simulation, after about 5000 ns (Figure 4c), all RLs are located inside the bilayer and none remain in the water.

It is important to note that the final location of the RLs is virtually the same as in the case when the RLs and lipids are co-solubilized (Figure 2c). The RL location is then independent of the starting configuration but rather reflects their most favorable position when they interact with phospholipids. It should be noticed that co-solubilizing lipids and RLs allows to reach the thermodynamic equilibrium forty-fold faster compared to RL addition to a pre-formed bilayer. Indeed, more than 5.5 µs are needed in the adding case and 130 ns in the co-solubilization case to reach the equilibrium. This validates the experimental use of co-solubilization techniques for liposome formation.

Given their amphiphilic nature, RLs are able to self-aggregate [4,24,50]. Our data confirm RL aggregation behavior (Figure 3b and Supplementary Materials Video S1). The MD simulations show that once an RL aggregate is formed, a single RL may then exits the aggregate and enters the lipid bilayer leading to the sporadic and non-collaborative insertion observed.

#### 2.2.2. Impact of RL Presence on Lipid Chain Dynamic

Rhamnolipid spontaneous interaction with plant plasma membrane phospholipids and their localization inside the bilayer raises the possibility of a lipid dynamic modification. Solid-state NMR spectroscopy is the method of choice to study the modifications induced by an external molecule on membrane model dynamics [35,47]. Indeed, the use of deuterium nucleus informs on the hydrophobic core dynamic using chain deuterated lipids [51,52,53]. Here, PLPC/POPC–^2^H_31_ (1/1) liposomes were compared to RL containing liposomes with a ratio of 1 RL for 50 lipids.

**Figure 5:**
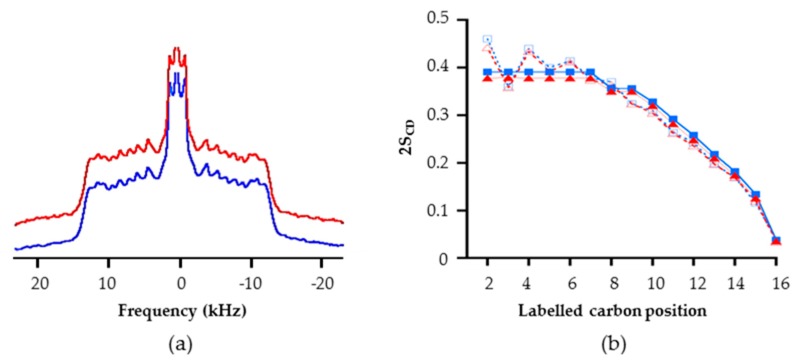
Study of RL mix impact on the dynamics of a PLPC/POPC–^2^H_31_ (1:1) membrane model at a RL/lipid ratio of 1:50 (**a**) and ^2^H NMR spectra in absence (in blue) and in presence (in red) of RLs (**b**). Order parameter, 2S_CD_, as a function of the labeled carbon position from the same experiment obtained by solid-state NMR spectroscopy (solid line) and molecular dynamics simulation of RL addition to a pre-equilibrated bilayer (dot line). (■) POPC/PLPC (NMR); (▲) POPC/PLPC + RLs (NMR); (□) POPC/PLPC (MD simulation); (Δ) POPC/PLPC + RLs (MD simulation).

In each case, a symmetric spectrum characteristic of lamellar fluid phase l_d_ was obtained (Figure 5a) [51,54]. No significant differences can be observed when comparing models with or without RLs. In order to access more accurately the dynamic of the hydrophobic core of the bilayer (cf. the deuterated palmitic chain of POPC), the first order parameters (S_CD_) were calculated from the NMR spectra in order to characterize the C–H bond fluctuations. The S_CD_ values can range from 0 to 1; with 1 corresponding to highly dynamic positions [54,55]. A diminution of S_CD_ values induced by an exogenous compound traduces an increase of lipid dynamics, and thus a potential destabilization. A perfect superposition of the profiles with and without RLs was obtained (Figure 5b, in order to increase graphic legibility 2S_CD_ are represented) [52,55]. For positions corresponding to the carbon numbers 2 to 7, the fluctuations of C–^2^H bonds were low and similar due to the rigidity provoked by the nearby glycerol group (i.e., “plateau” region in Figure 5b), which induces higher S_CD_ values. On the contrary, at the center of the bilayers, local dynamic were more fluid with faster C–^2^H bond fluctuation decreasing S_CD_ values. These results suggest that RLs do not strongly disturb the dynamic of this phospholipid membrane model. They were confirmed by experiments carried out at the acidic pH of the plant apoplast (5,5 [56]) with RL/lipid ratios ranging from 1:50 to 1:10 (Supplementary Materials Figure S1 and Table S1). The first spectral moment (M_1_) which quantify globally membrane dynamic was not affected by RL presence on a temperature scale from 10 °C to 38 °C (Supplementary Materials Figure S2).

The S_CD_ were also determined from MD simulation of RLs into POPC/PLPC bilayers. The S_CD_ were computed for positions 2 to 16 of the palmitic chain of POPC before and after RL insertion. For positions 2 to 8, 2S_CD_ values around 0.4 were obtained (Figure 5b). Then a clear decrease was observed from position 9 to 16 with a final value of 0.025. As S_CD_ values obtained with and without RLs coincide, POPC acyl chains dynamic appeared to be unaffected by insertion of RLs. The RL dispersion into phospholipids seemed to allow them to stay in the biomimetic membrane without affecting the dynamic of lipids. Together, MD and NMR data pointed to a non-disturbing insertion of RLs into PC bilayers. This result appears consistent with FTIR data presented in Figure 2 and the absence of modification of CH_3_ and CH_2_ group absorbance. Moreover, the effects observed on C=O and P=O in FTIR experiments, i.e., a diminution of the hydrogen-bonded state of these groups, suggest a lower fluidity in the polar head space without impacting global membrane dynamics.

### 2.3. Getting Closer to Lipid Composition of Plant Plasma Membrane

The biological membrane lipid part was composed of a large diversity of phospholipids, sterols, and sphingolipids. Regarding this complexity, the presented lipid bilayer models, although well-proven for biophysical studies, remain primary models to mimic plant plasma membranes. To get closer to biological reality, RL effect was characterized on more complex biomimetic membranes. To highlight an eventual modification of the NMR spectral shape, a RL/lipid ratio of 1:25 was used.

In order to take into account: (i) the presence of β-sitosterol, the main sterol in *A. thaliana*; (ii) the presence of sphingolipids, (iii) the presence of anionic lipids, and (iv) the high diversity of phospholipids and acyl chains found in the target membrane, a soy PC/POPE–^2^H_31_/soy phosphatidylinositol (PI)/soy phosphatidylglycerol (PG)/β-sitosterol/soy glucosylceramide (2.6/2.5/0.6/0.6/3.2/0.5) representative plant lipid model was carried out. All lipid proportions were calculated based on the *A. thaliana* plasma membrane lipid composition [45,57,58].

**Figure 6:**
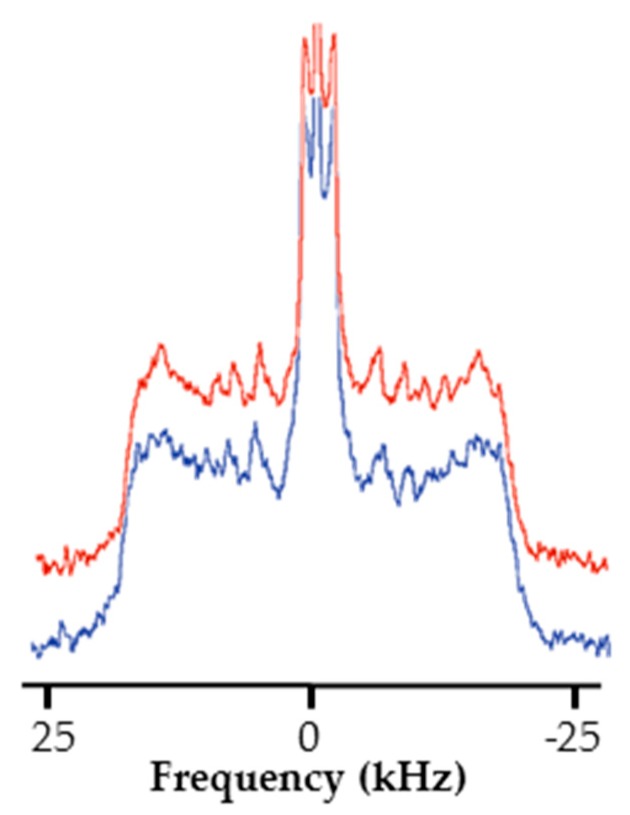
RL mix impact on the dynamic of a soy PC/POPE–^2^H_31_/soy PI/soy PG/β-sitosterol/soy glucosylceramide (2.6/2.5/0.6/0.6/3.2/0.5) plant model at a RL/lipid ratio of 1:25. ^2^H NMR spectra in absence (in blue) and in presence of RLs (in red).

The NMR spectra of this representative model are presented in absence (Figure 6) and in the presence of RLs (Figure 6). An increase of the spectral width compared to those observed in Figure 5 for the simple model can be noticed. This can be explained by the presence of β-sitosterol causing the emergence of a sterol-enhanced phase called liquid-ordered phase l_o_ [59,60]. Furthermore, whatever the insertion of RLs, there was no significant difference between spectra. Once more, this may suggest that RLs do not disturb lipid dynamics. Nevertheless, it should be noticed that a drastic loss in spectrum resolution with this model leads to a loss of information preventing a deeper study. Similar results were obtained for experiments realized at the plant apoplastic pH (Supplemental Maerials Figure S3).

The observed loss of resolution cannot be imputed to sterol as well-resolved spectra were already obtained in the presence of sterol for binary or ternary lipid models [47,61,62,63]. It has already been reported that low-resolved spectra may be due to the high complexity of the systems [9,55]. The S_CD_ calculation through MD experiments could be an interesting possibility to overcome this problem but would demand, in this case, an important investment in plant specific lipid topology realization. Altogether, our results on plant plasma membrane models suggest that the insertion of RLs inside plant plasma membrane do not strongly destabilize it, and that the effect on plant lipid dynamic is very subtle. Still specific interactions with other plant lipids like glycosylinositol phosphorylceramides or conjugates phytosterols could modify this equilibrium [64]. Unfortunately, the commercial availability of sufficient purified amounts of those lipids is lacking to build biomimetic membranes.

### 2.4. RL Effect on a Fungi Membrane Model Dynamic

In fungi, RLs have been shown to have negative effects such as mycelial growth inhibition, spore germination inhibition, and zoospore lysis [7]. Those activities are supposed to be a consequence of RL interaction with lipids, but data on specific fungi model membrane are lacking [8,21,65]. Here we studied the interaction of RLs with a fungal membrane model. This model is a compromise between two main concerns. On one hand, there is the biological representativeness of fungus membrane composition. We considered the RL sensitive fungus *B. cinerea*, and took into account the presence in its membrane of high amounts of ergosterol and the high ratio of anionic/zwitterionic phospholipids [24,66,67]. On the other hand, we took into account the need of a limited complexity and sterol content to obtain well-resolved liposome spectra [9,55]. From those elements, a model containing 53% of the deuterated POPC–^2^H_31_, 22% of the anionic and unsaturated 1-palmitoyl-2-oleoyl-sn-glycero-3-phospho-(1′-rac-glycerol) (POPG), and 25% of ergosterol was built. As previously noted, a ratio of 1 RL for 25 lipids was used for RL containing liposomes.

**Figure 7:**
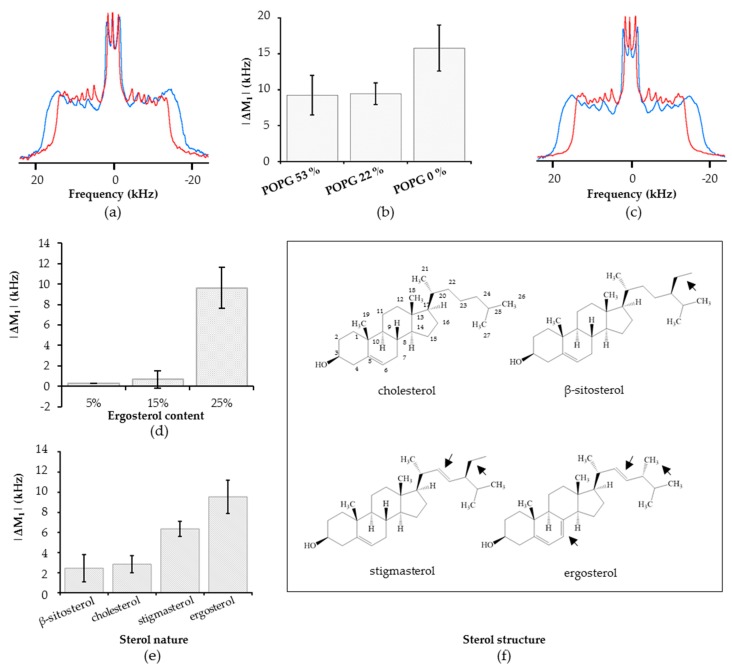
^2^H NMR study of RL mix effect on ternary POPC–^2^H_31_/POPG/sterol models. (**a**) Spectra of POPC–^2^H_31_/POPG/ergosterol (53/22/25) in the absence (in blue) and in the presence (in red) of RLs; (**b**) first spectral moment variation (|ΔM_1_|), induced by RL presence for the models POPC–^2^H_31_/POPG/ergosterol (22/53/25), (53/22/25) and (75/0/25); (**c**) spectra of POPC–^2^H_31_/ergosterol (75/25) in the absence (in blue) and in the presence (in red) of RLs; (**d**) |ΔM_1_| induced by RL presence for the models POPC–^2^H_31_/POPG/ergosterol (53/22/25), (60/25/15), and (67/28/5); (**e**) |ΔM_1_| induced by RL presence for POPC–^2^H_31_/POPG/sterol models, with respectively β-sitosterol, cholesterol, stigmasterol, and ergosterol; (**f**) structures of the different sterols used.

The insertion of RLs has a clear impact on spectra obtained on the POPC–^2^H_31_/POPG/ergosterol model. A decrease of the spectral width from 31.4 to 27.4 kHz in the presence of RLs was observed (Figure 7a). The change was obtained on all quadripolar splitting, including those in the middle of the spectra corresponding to positions of deuterated lipids in the acyl core of the membrane. The overall decreases in spectral width correspond to an increase in the dynamic of the membrane hydrophobic core. This conclusion was also supported by the observation of a clear M_1_ decrease (|ΔM_1_|) of 9.55 ± 1.65 kHz due to RL presence, traducing a global enhancement of membrane dynamic (Figure 7b).

This model differs from previously presented plant plasma membrane models on two main points: the presence of a high amount of anionic lipids and the presence of the fungal sterol, ergosterol. In order to investigate the involvement of anionic lipids in the RL-induced dynamic modification, a model with higher POPG ratio and one without POPG were investigated. Those models were compared through calculation of M_1_ variations induced by RLs. Rhamnolipids induced very similar shifts on the POPC–_2_H^31^/POPG/ergosterol (53/22/25) and (22/53/25) model as shown by the very similar |ΔM_1_| (Figure 7b). Interestingly, a greater fluidization was induced by RLs on the model without anionic lipids (Figure 7b). In that case, the spectral width was reduced from 33.6 to 27.4 kHz in the presence of RLs (Figure 7c).

From those results, it appears that POPG presence was not decisive for RL-induced fluidization and that the presence of ergosterol was probably the key factor to explain the effect on membrane dynamics. To test this hypothesis, two other models with reduced ergosterol contents and identical anionic/zwitterionic lipid ratio were used: POPC/POPG/ergosterol (60/25/**15**) and (67/28/**5**). The impact of RLs was dramatically reduced compared to the POPC–^2^H_31_/POPG/ergosterol (53/22/**25**) model as shown by the strong decrease of |ΔM_1_| from 9 ± 1.65 kHz to 0.6 ± 0.80 and 0.3 ± 0.01 kHz for respectively 25, 15, and 5% ergosterol content models (Figure 7d). This result shows an obvious link between RL-induced fluidization and high ergosterol content. To confirm this result, copies of the fungal model were realized replacing ergosterol by either the mammal sterol cholesterol or one of the phytosterols β-sitosterol and stigmasterol. On those three models, the enhancement of membrane fluidity induced by RLs was reduced as compared to the observations made on the ergosterol corresponding model (Figure 7e). A clear enhancement in lipid dynamic of the stigmasterol containing model was still observed in presence of RLs, with a |ΔM_1_| of 6.35 ± 0.75 kHz. In contrast, β-sitosterol and cholesterol containing models were only slightly affected with |ΔM_1_| of 2.45 ± 1.35 and 2.85 ± 0.85 respectively.

Here, it clearly appears that RLs enhance lipid dynamic on the fungal model membrane considered. This fluidity increase appears to be uncorrelated to the presence of anionic lipids but is directly dependent of sterol amount and nature, ergosterol containing model membranes being the more affected ones. It can be suspected that, in vivo, sterol rich membrane domains present in plants and fungi are more affected than others by RLs presence [68,69]. Besides, the impact of sterol nature is striking as those molecules are structurally somehow similar (Figure 7f). The two sterols involved in RL-affected models are ergosterol and stigmasterol which are distinct by only two points: the presence of a double bond between the carbons 7 and 8 on ergosterol and the nature and the stereochemistry of the alkyl group on position 24. Those two differences could be at the origin of the |ΔM_1_| gap observed between stigmasterol and ergosterol containing models. The difference between the interaction of RLs with β-sitosterol- and stigmasterol-based models is particularly outstanding as those phytosterols only differ by one double bond between carbons 22 and 23. Indeed, in *A. thaliana*, those two sterols are produced by the same pathway, β-sitosterol being converted in stigmasterol [70]. Interestingly, this double bond is present in both stigmasterol and ergosterol, but neither in β-sitosterol and cholesterol. So, it could be hypothesized from our study that the ability of RLs to trigger membrane fluidization correlates with the presence of a sterol containing this double bond. Specific interaction due to small structural differences in sterols, leading to significant effect on membrane with RLs, could be more precisely described by MD simulations This requires having the sterol topology description in the Force Field used, which is not available currently.

Our data clearly shows that the fungal-membrane-based model dynamic was enhanced by RLs. This phenomenon could, in vivo, corresponds to a destabilization of fungal plasma membrane at the origin of RL antifungal activity. An interaction of RLs with ergosterol as a molecular origin of RLs’ antifungal activity appears plausible as ergosterol is the target of numerous natural (and chemical) antibacterial compounds, including some plant proteins and metabolites involved in defense responses [71,72]. The absence of an effect on a cholesterol containing model can be noted but does not rule out the possibility of different interactions with mammal specific models. In plant plasma membranes, the presence of mixed phytosterols could be associated to a moderated destabilization, as models containing β-sitosterol are not affected, to the contrary of stigmasterol containing models. Interestingly, plants known to perceive RLs, *A. thaliana*, grapevine, and *B. napus*, have β-sitosterol as the main sterol and smaller amounts of stigmasterol in controlled conditions [73,74,75,76]. A small enhancement of lipid dynamic, potentially induced by RL interaction with stigmasterol, could slightly destabilize plant plasma membranes. This modification could be more pronounced in sterol-rich membrane domains, which are known to be implicated in the activation and/or the regulation of some defense related membrane proteins [77,78]. There, RL-induced subtle changes could trigger defense responses, by modifying the dynamic of membrane proteins surrounding lipids. If our work does not exclude the existence of a specific proteic receptor involved in RL perception in plants, it highlights the possibility of a direct lipid-driven process. The study of mutants affected in lipid membrane composition could be a key step to assess this hypothesis in vivo.

## 3. Materials and Methods

### 3.1. Materials

Most lipids used, POPC, POPC–^2^H_31_, POPE–^2^H_31_, POPG, PLPC, soy glucosylceramide, soy PC, soy PG, soy PI, β-sitosterol, stigmasterol, and cholesterol were purchased from Avanti Polar Lipids (Alabaster, Al, USA). Ergosterol was from the Cayman Chemical Company (Ann Arbor, MI, USA). The RL mixture was composed of 40% of α-l-rhamnopyranosyl-β-hydroxydecanoyl-β-hydroxydecanoate and 60% of 2-*O*-α-l-rhamnopyranosyl-α-l-rhamnopyranosyl-β-hydroxydecanoyl-β-hydroxydecanoate from *P. aeruginosa* secretome (Jeneil, Saukville, WI, USA) and purified up to 99% as previously described [23]. The average molar mass is 591 g mol^−1^ given the mono-RL/di-RL proportion. When needed, separation of mono-RLs and di-RLs were realized by high-performance liquid chromatography coupled to an evaporative light scattering detector (HPLC–ELSD) on an Interchim Uptisphere Strategy C18-2 column (21.2 mm, 15 µm) on an Interchim Puriflash 4250 system. Before injection, the mix was solubilized in pure methanol and filtered through a 0.22-µm polytetrafluoroethylene (PTFE) membrane. Distilled water (0.1 %, *v*/*v*, of formic acid) and acetonitrile (ACN) (0.1 %, *v*/*v*, of formic acid) were used as mobile phase. For the first 8 min, the percentage of ACN was increased from 60% to 100%. Pure ACN was then used for 8 min. The percentage of ACN was decreased to 60% in 30 sec and the column was cleaned during 3 min. The flow was 20 mL min^−1^. The purity of the collected fractions was checked by HPLC-ELSD. The ELSD parameters were 35 °C and 2.5 bar. Pure fractions were then pooled and dried with a vacuum apparatus (10 mbar, 40 °C).

Analytical quality solvents were used to solubilize lipids and RLs were purchased from Fisher Scientific (Illkirch-Graffenstaden, France) and Sigma–Aldrich (Saint Quentin Fallavier, France). Sodium salt, 2-amino-2-(hydroxymethyl)propane-1,3-diol (Tris) and 2,2-Bis(hydroxymethyl)-2,2′,2″-nitrilotriethanol (Bis-Tris) used for buffer solution preparation were also from Sigma–Aldrich. Ultrapure water, with a nominal resistivity of 18.2 MΩcm, was used for hydration, buffer preparation, and lyophilization.

### 3.2. FTIR Experiments

#### 3.2.1. Sample Preparation

The FTIR experiments were conducted on pure PLPC liposomes. Lipids were dissolved in a chloroform/methanol mixture (2/1 *v*/*v*) and supplemented with mono- or di-RLs when necessary, at a molar lipid/RL ratio of 19:1. The solvent mixture was then evaporated before sample hydration with D_2_O. Continuous mixing was realized to obtain multilamellar vesicles.

#### 3.2.2. Data Acquisition and Analysis

Experiments were carried out on a Bruker Equinox 55 spectrometer (Karlsruhe, Germany) equipped with a liquid-nitrogen-cooled DTGS (deuterated tri glycine sulfate) detector, and continuously purged with N_2_ during data acquisition. For each experiment, 128 scans were realized with a 4 cm^−1^ resolution at room temperature A demountable cell (Bruker) equipped with CaF_2_ windows was used for all experiments. At least two independent measurements were realized to assure results accuracy.

### 3.3. Molecular Dynamics Simulations

All MD simulations were performed using the GROMACS suite [79,80]. Maestro software (Schrödinger, LLC, New York, NY, USA) was used to build atomistic versions of deprotonated mono- and di-RL that were then submitted to the Automated Topology Builder website [81,82] in order to obtain a basic topology. These ATB-built topologies were manually refined in order to be more compliant with the Slipids forcefield [83] that was used for simulations. In particular, Slipids parameters were used to describe the aliphatic carbons from both RLs as this forcefield simulates lipid chain order parameters well [84]. The sugar moiety was described by the means of GLYCAM06 forcefield [85] in conjunction with AMBER ff99SB-ILDN [85]. The TIP3P model [86] was used to describe water molecules. Smooth particle mesh Ewald (SPME) method [87] with a direct-to-Fourier-space cutoff of 1.0 nm was used for electrostatics along with a 1.0 nm van der Waals cutoff. V-rescale thermostat [88] was chosen to maintain the systems at 300 K with a 0.1 fs time constant. All bonds were constrained using the LINear Constraint Solver (LINCS) algorithm [89]. In order to let water molecules relax, systems were first simulated for 1 ns in the isovolumic NVT ensemble followed by 1 ns in the isothermal–isobaric (NPT) ensemble; in both cases, only water molecules were not position-restrained. Parrinello–Rahman barostat [90,91] was used to maintain pressure to 1.013 bar semi-isotropically (*z*-axis pressure being uncoupled to XY plane) with a time constant of 10 ps. Finally, systems were simulated without any restraints in the NPT ensemble during a production run in order to accumulate data for analysis. All MD trajectory were analyzed using GROMACS and MDAnalysis [92,93]. Visualization was done thanks to VMD [94,95].

Two different systems were simulated. In the first one, 4 mono-RLs, 6 di-RLs, 256 POPC, and 256 PLPC (lipid/RL ratio of 51) were randomly inserted in a box. The box was then hydrated using 29523 water molecules (i.e., around 58 water molecules per lipid) and 10 Na^+^ ions (for neutrality). This system, which mimic the co-solubilization done to prepare experimental samples, was equilibrated (vide supra) and simulated for 300 ns. The whole procedure was performed twice to ensure reproducibility. Both simulations leading to virtually the same results, only one was presented here. To simulate the insertion of RLs inside a lipid bilayer, a second system with the same composition as the first one was simulated. As opposed to the first system, here the RLs were randomly added above and below an already-equilibrated POPC/PLPC bilayer. Then the same number of water and Na^+^ ions were added. After a short equilibration, this second system was simulated for 7000 ns, until the system was completely equilibrated, and no further evolution was observed. Given its long duration, this simulation was performed only once but other smaller (1 RL, 128 lipids) and shorter simulations (300 ns) gave quite similar results (data not shown).

### 3.4. NMR Experiments

#### 3.4.1. Sample Preparation

The liposomes used in the solid-state NMR experiments were carried out according to the conventional protocol described hereafter. Lipids were solubilized in chloroform at a fixed concentration of 10 mg mL^−1^ for sterols and of 25 mg mL^−1^ for phospholipids. Lipid solutions were mixed in order to obtain the right proportions in a total lipid amount of 0.012 mmol. If needed, 50 or 100 µL of a 2.84 mg mL^−1^ RL mix solution were added to obtain a RL/lipid ratio of respectively 1:50 or 1:25. The resulting solution was evaporated under nitrogen gas flow to obtain a thin lipid film. The sample was hydrated with ultrapure water, well-vortexed to promote a total hydration of the film and lyophilized overnight to remove the last traces of solvents. The resulting powder containing lipids and RLs was hydrated by 80 µL of salt buffer solution (100 mM of sodium salt with 25 mM of Tris or Bis-Tris for pH 7.5 or 5.5, respectively), vortexed (2 min, 3000 rpm) and homogenized using four freeze-thaw cycles involving one step of freezing (−80 °C, 15 min) following by thawing (40 °C, 10 min) and shaking (3000 rpm, 45 s). Finally, a milky fluid suspension of micrometer size multilamellar vesicles was obtained at a lipid concentration of 150 mM. Those samples were inserted in a 7-mm solid-state NMR rotor to analyze. All experiments were done at least twice in order to have replicates.

#### 3.4.2. Data Acquisition and Analysis

All experiments were carried out on a Bruker Avance Biospin 300 WB (7.05 T) equipped with a CP-MAS 7-mm probe. First ^2^H NMR spectra of PLPC/POPC–^2^H_31_ (1/1), POPC/POPC–^2^H_31_ (1/1), and soy PC/POPE–^2^H_31_/soy PI/soy PG/β-sitosterol/soy glucosylceramide (2.6/2.5/0.6/0.6/3.2/0.5) were recorded varying the temperature from 10 to 38 °C using an increment of 4 °C. Before each acquisition, samples were allowed 30 min to equilibrate at defined temperature. For this study, temperature calibration of the probe was performed previously using methanol–^2^H_4_/methanol (96/4) and ethylene glycol/DMSO–^2^H_6_ (80/20) samples according to a standard protocol [96]. In each case, a small decrease of spectral width was noted with increasing temperature. This diminution reflects the increase of global lipids dynamics caused by the augmentation of temperature (e.g., 52, 45.5, and 40.5 MHz for PLPC/POPC–^2^H_31_ (1/1) alone at 10, 26, and 38 °C, respectively) and no phase transition was observed for any model. For all following experiments, a reference temperature of 26 °C was chosen, close to room temperature and to FTIR and MD experiment temperatures.

The ^2^H NMR experiments were carried out using a phase cycled quadrupolar echo pulse sequence (90°*x*-τ-90°*y*-τ-acq) [97]. Parameters used for the ^2^H NMR experiments are listed below: spectral width of 150 kHz, π/2 pulse delays of 5.25 µs, an interpulse delay of 40 µs, a recycled delay of 1.5 s, and a number of acquisitions ranging from 8 k to 14 k depending on samples. For all spectra, an exponential line broadening of 100 Hz was applied before Fourier transform from the top of the echo. First spectral moment (M_1_) determination was realized with NMR depaker (unpublished material [98]). |ΔM_1_| were calculated as the absolute value of the difference between RL containing and reference sample spectra M_1_. For order parameters (S_CD_) calculation, spectra were simulated using “multisca” (E. J. Dufourc, unpublished material) in order to determine quadripolar splittings (${\Delta \upsilon }_{Q}^{0{}^{\circ}}$) for each C–^2^H bond. The S_CD_ calculation was then carried out using Equation (1), with $A_{Q}$ static deuterium quadrupolar coupling constant (167 kHz for C–^2^H bonds) [99].

(1) $${\Delta \upsilon }_{Q}^{0{}^{\circ}}=\frac{3}{2}A_{Q}S_{CD}$$

## 4. Conclusions

From our results it can be proposed that RLs interact with plasma membrane models. They spontaneously insert through monomeric forms into different membrane models to localize near the phospholipid glycerol backbone. They have a limited impact on the dynamic of phospholipid chains but enhance the fluidity of some sterol containing models, with a composition dependent effect. In fungi, a destabilization of the membrane due to ergosterol content could lead to deleterious effects. In plants, the phytosterol content could explain a subtler effect. Globally, our results support the hypothesis of a membrane destabilization driven antifungal activity of RLs, but suggest that more complex interactions between RL and membrane can be involved in their perception by plant.

## Supplementary Materials

Supplementary materials can be found at https://www.mdpi.com/1422-0067/20/5/1009/s1.

## Abbreviations

ACN	acetonitrile
DMPC	1,2-dimyristoyl-sn-glycero-3-phosphocholine
DPPC	1,2-dipalmitoyl-sn-glycero-3-phosphocholine
DTGS	deuterated tri glycine sulfate
ELSD	evaporative light scattering detector
ERDF	European regional development fund
FTIR	Fourier transform infrared spectroscopy
HdF	Hauts de France
HPLC	high-performance liquid chromatography
LINCS	linear constraint solver
M_1_	first spectral moment
MD	molecular dynamics
NMR	nuclear magnetic resonance
PC	phosphatidylcholine
PE	phosphatidylethanolamine
PG	phosphatidylglycerol
PI	phosphatidylinositol
PLPC	1-palmitoyl-2-linoleoyl-sn-glycero-3-phosphocholine
POPC	1-palmitoyl-2-oleoyl-glycero-3-phosphocholine
POPG	1-palmitoyl-2-oleoyl-sn-glycero-3-phospho-(1′-rac-glycerol)
PTFE	polytetrafluoroethylene
RL	rhamnolipid
S_CD_	notation for the first order parameter
SPMES_CD_	smooth particle mesh Ewaldfirst order parameter

## References

[1] Abdel-Mawgoud A.M., Lépine F., Déziel E. (2010). Rhamnolipids: Diversity of structures, microbial origins and roles. Appl. Microbiol. Biotechnol..

[2] Randhawa K.K.S., Rahman P.K.S.M. (2014). Rhamnolipid biosurfactants-past, present, and future scenario of global market. Front. Microbiol..

[3] Santos D.K.F., Rufino R.D., Luna J.M., Santos V.A., Sarubbo L.A. (2016). Biosurfactants: Multifunctional Biomolecules of the 21st Century. Int. J. Mol. Sci..

[4] Ishigami Y., Gama Y., Nagahora H., Yamaguchi M., Nakahara H., Kamata T. (1987). The pH-sensitive conversion of molecular aggregates of rhamnolipid biosurfactant. Chem. Lett..

[5] Kłosowska-Chomiczewska I.E., Mędrzycka K., Hallmann E., Karpenko E., Pokynbroda T., Macierzanka A., Jungnickel C. (2017). Rhamnolipid CMC prediction. J. Colloid Interface Sci..

[6] Cieśla J., Koczańska M., Bieganowski A. (2018). An interaction of rhamnolipids with Cu^2+^ ions. Molecules.

[7] Vatsa P., Sanchez L., Clément C., Baillieul F., Dorey S. (2010). Rhamnolipid biosurfactants as new players in animal and plant defense against microbes. Int. J. Mol. Sci..

[8] Chen J., Wu Q., Hua Y., Chen J., Zhang H., Wang H. (2017). Potential applications of biosurfactant rhamnolipids in agriculture and biomedicine. Appl. Microbiol. Biotechnol..

[9] Deleu M., Crowet J.M., Nasir M.N., Lins L. (2014). Complementary biophysical tools to investigate lipid specificity in the interaction between bioactive molecules and the plasma membrane: A review. Biochim. Biophys. Acta Biomembr..

[10] Pashynska V.A. (2009). Mass spectrometric study of rhamnolipid biosurfactants and their interactions with cell membrane phospholipids. Biopolym. Cell.

[11] Aranda F.J., Espuny M.J., Marque A., Teruel J.A., Manresa A., Ortiz A. (2007). Thermodynamics of the Interaction of a Dirhamnolipid Biosurfactant Secreted by *Pseudomonas aeruginosa* with Phospholipid Membranes. Langmuir.

[12] Sánchez M., Aranda F.J., Teruel J.A., Espuny M.J., Marqués A., Manresa A., Ortiz A. (2010). Permeabilization of biological and artificial membranes by a bacterial dirhamnolipid produced by *Pseudomonas aeruginosa*. J. Colloid Interface Sci..

[13] Ortiz A., Teruel J.A., Espuny M.J., Marqués A., Manresa A., Aranda F.J. (2006). Effects of dirhamnolipid on the structural properties of phosphatidylcholine membranes. Int. J. Pharm..

[14] Sánchez M., Aranda F.J., Teruel J.A., Ortiz A. (2009). Interaction of a bacterial dirhamnolipid with phosphatidylcholine membranes: A biophysical study. Chem. Phys. Lipids.

[15] Abbasi H., Noghabi K.A., Ortiz A. (2012). Interaction of a bacterial monorhamnolipid secreted by *Pseudomonas aeruginosa* MA01 with phosphatidylcholine model membranes. Chem. Phys. Lipids.

[16] Sánchez M., Aranda F.J., Teruel J.A., Ortiz A. (2011). New pH-sensitive liposomes containing phosphatidylethanolamine and a bacterial dirhamnolipid. Chem. Phys. Lipids.

[17] Haba E., Pinazo A., Pons R., Pérez L., Manresa A. (2014). Complex rhamnolipid mixture characterization and its influence on DPPC bilayer organization. Biochim. Biophys. Acta.

[18] Moussa Z., Chebl M., Patra D. (2017). Interaction of curcumin with 1,2-dioctadecanoyl-sn-glycero-3-phosphocholine liposomes: Intercalation of rhamnolipids enhances membrane fluidity, permeability and stability of drug molecule. Colloids Surf. B Biointerfaces.

[19] Sánchez M., Teruel J.A., Espuny M.J., Marqués A., Aranda F.J., Manresa A., Ortiz A. (2006). Modulation of the physical properties of dielaidoylphosphatidylethanolamine membranes by a dirhamnolipid biosurfactant produced by *Pseudomonas aeruginosa*. Chem. Phys. Lipids.

[20] Abbasi H., Aranda F.J., Noghabi K.A., Ortiz A. (2013). A bacterial monorhamnolipid alters the biophysical properties of phosphatidylethanolamine model membranes. Biochim. Biophys. Acta.

[21] Stanghellini M.E., Miller R.M. (1997). Biosurfactants: Their Identity and Potential Efficacy in the Biological Control of Zoosporic Plant Pathogens. Plant Dis..

[22] Kim B.S., Lee J.Y., Hwang B.K. (2000). In vivo control and in vitro antifungal activity of rhamnolipid B, a glycolipid antibiotic, against *Phytophthora capsici* and *Colletotrichum orbiculare*. Pest Manag. Sci..

[23] Varnier A.-L., Sanchez L., Vatsa P., Boudesocque L., Garcia-Brugger A., Rabenoelina F., Sorokin A., Renault J.-H., Kauffmann S., Pugin A. (2009). Bacterial rhamnolipids are novel MAMPs conferring resistance to *Botrytis cinerea* in grapevine. Plant Cell Environ..

[24] Monnier N., Furlan A., Botcazon C., Dahi A., Mongelard G., Cordelier S., Clément C., Dorey S., Sarazin C., Rippa S. (2018). Rhamnolipids From *Pseudomonas aeruginosa* Are Elicitors Triggering *Brassica napus* Protection Against *Botrytis cinerea* Without Physiological Disorders. Front. Plant Sci..

[25] Sanchez L., Courteaux B., Hubert J., Kauffmann S., Renault J.-H., Clément C., Baillieul F., Dorey S. (2012). Rhamnolipids elicit defense responses and induce disease resistance against biotrophic, hemibiotrophic, and necrotrophic pathogens that require different signaling pathways in *Arabidopsis* and highlight a central role for salicylic acid. Plant Physiol..

[26] Yan F., Hu H., Lu L., Zheng X. (2015). Rhamnolipids induce oxidative stress responses in cherry tomato fruit to *Alternaria alternata*. Pest Manag. Sci..

[27] Ranf S. (2017). Sensing of molecular patterns through cell surface immune receptors. Curr. Opin. Plant Biol..

[28] Bauer J., Brandenburg K., Zähringer U., Rademann J. (2006). Chemical Synthesis of a Glycolipid Library by a Solid-Phase Strategy Allows Elucidation of the Structural Specificity of Immunostimulation by Rhamnolipids. Chem. Eur. J..

[29] Kouzayha A., Wattraint O., Sarazin C. (2009). Interactions of two transmembrane peptides in supported lipid bilayers studied by a ^31^P and ^15^N MAOSS NMR strategy. Biochimie.

[30] Rippa S., Eid M., Formaggio F., Toniolo C., Béven L. (2010). Hypersensitive-like response to the pore-former peptaibol alamethicin in *Arabidopsis thaliana*. Chembiochem.

[31] Haapalainen M., Engelhardt S., Küfner I., Li C.M., Nürnberger T., Lee J., Romantschuk M., Taira S. (2011). Functional mapping of harpin HrpZ of *Pseudomonas syringae* reveals the sites responsible for protein oligomerization, lipid interactions and plant defence induction. Mol. Plant Pathol..

[32] Guan X., Buchholz G., Nick P. (2013). The cytoskeleton is disrupted by the bacterial effector HrpZ, but not by the bacterial PAMP flg22, in tobacco BY-2 cells. J. Exp. Bot..

[33] Henry G., Deleu M., Jourdan E., Thonart P., Ongena M. (2011). The bacterial lipopeptide surfactin targets the lipid fraction of the plant plasma membrane to trigger immune-related defence responses. Cell. Microbiol..

[34] Nasir M.N., Lins L., Crowet J.M., Ongena M., Dorey S., Dhondt-Cordelier S., Clément C., Bouquillon S., Haudrechy A., Sarazin C. (2017). Differential Interaction of Synthetic Glycolipids with Biomimetic Plasma Membrane Lipids Correlates with the Plant Biological Response. Langmuir.

[35] Luzuriaga-Loaiza W.P., Schellenberger R., De Gaetano Y., Obounou Akong F., Villaume S., Crouzet J., Haudrechy A., Baillieul F., Clément C., Lins L. (2018). Synthetic Rhamnolipid Bolaforms trigger an innate immune response in *Arabidopsis thaliana*. Sci. Rep..

[36] Buchoux S., Lai-Kee-Him J., Garnier M., Tsan P., Besson F., Brisson A., Dufourc E.J. (2008). Surfactin-triggered small vesicle formation of negatively charged membranes: A novel membrane-lysis mechanism. Biophys. J..

[37] Heerklotz H., Wieprecht T., Seelig J. (2004). Membrane Perturbation by the Lipopeptide Surfactin and Detergents as Studied by Deuterium NMR. J. Phys. Chem. B.

[38] Kell H., Holzwarth J.F., Boettcher C., Heenan R.K., Vater J. (2007). Physicochemical studies of the interaction of the lipoheptapeptide surfactin with lipid bilayers of L-alpha-dimyristoyl phosphatidylcholine. Biophys. Chem..

[39] Grau A., Fernández J.G., Peypoux F., Ortiz A. (1999). A study on the interactions of surfactin with phospholipid vesicles. Biochim. Biophys. Acta.

[40] Liu J., Hagberg I., Novitsky L., Hadj-Moussa H., Avis T.J. (2014). Interaction of antimicrobial cyclic lipopeptides from *Bacillus subtilis* influences their effect on spore germination and membrane permeability in fungal plant pathogens. Fungal Biol..

[41] Lebrón-Paler A., Pemberton J.E., Becker B.A., Otto W.H., Larive C.K., Maier R.M. (2006). Determination of the acid dissociation constant of the biosurfactant monorhamnolipid in aqueous solution by potentiometric and spectroscopic methods. Anal. Chem..

[42] Abdel-Mawgoud A.M., Aboulwafa M.M., Hassouna N.A.-H. (2009). Characterization of Rhamnolipid Produced by *Pseudomonas aeruginosa* Isolate Bs20. Appl. Biochem. Biotechnol..

[43] Wang H., Coss C.S., Mudalige A., Polt R.L., Pemberton J.E. (2013). A PM-IRRAS investigation of monorhamnolipid orientation at the air-water interface. Langmuir.

[44] Salnikov E.S., Mason A.J., Bechinger B. (2009). Membrane order perturbation in the presence of antimicrobial peptides by ^2^H solid-state NMR spectroscopy. Biochimie.

[45] Uemura M., Joseph R.A. (1995). Cold Acclimation of *Arabidopsis Thaliana*. Effect on Plasma Membrane Lipid Composition and Freeze-lnduced Lesions. Plant Physiol..

[46] Goni F.M., Arrondo J.L.R. (1986). A study of phospholipid phosphate groups in model membranes by Fourier transform IR spectroscopy. Faraday Discuss. Chem. Soc..

[47] Mason A.J., Marquette A., Bechinger B. (2007). Zwitterionic Phospholipids and Sterols Modulate Antimicrobial Peptide-Induced Membrane Destabilization. Biophys. J..

[48] Svetlovics J.A., Wheaten S.A., Almeida P.F. (2012). Phase separation and fluctuations in mixtures of a saturated and an unsaturated phospholipid. Biophys. J..

[49] Lee D.K., Bhunia A., Kotler S.A., Ramamoorthy A. (2015). Detergent-Type Membrane Fragmentation by MSI-78, MSI-367, MSI-594, and MSI-843 Antimicrobial Peptides and Inhibition by Cholesterol: A Solid-State Nuclear Magnetic Resonance Study. Biochemistry.

[50] Chen M.L., Penfold J., Thomas R.K., Smyth T.J.P., Perfumo A., Marchant R., Banat I.M., Stevenson P., Parry A., Tucker I. (2010). Solution Self-Assembly and Adsorption at the Air−Water Interface of the Monorhamnose and Dirhamnose Rhamnolipids and Their Mixtures. Langmuir.

[51] Davis J.H. (1983). The description of membrane lipid conformation, order and dynamics by ^2^H-NMR. Biochim. Biophys. Acta Rev. Biomembr..

[52] Brown M., Lope-Piedrafita S., Webb G.A. (2006). Solid-State Deuterium NMR Spectroscopy of Membranes.

[53] Grélard A., Loudet C., Diller A., Dufourc E.J., Lacapère J.-J. (2010). NMR Spectroscopy of Lipid Bilayers. Membrane Protein Structure Determination: Methods and Protocols.

[54] Davis J.H., Maraviglia B., Weeks G., Godin D.V. (1979). Bilayer rigidity of the erythrocyte membrane ^2^H-NMR of a perdeuterated palmitic acid probe. Biochim. Biophys. Acta.

[55] Leung S.S.W., Thewalt J., Separovic F., Naito A. (2014). Deuterium NMR of Mixed Lipid Membranes.

[56] Felle H.H. (2001). pH: Signal and Messenger in Plant Cells. Plant Biol..

[57] Minami A., Fujiwara M., Furuto A., Fukao Y., Yamashita T., Kamo M., Kawamura Y., Uemura M. (2009). Alterations in detergent-resistant plasma membrane microdomains in *Arabidopsis thaliana* during cold acclimation. Plant Cell Physiol..

[58] Funnekotter B., Kaczmarczyk A., Turner S.R., Bunn E., Zhou W., Smith S., Flematti G., Mancera R.L. (2013). Acclimation-induced changes in cell membrane composition and influence on cryotolerance of in vitro shoots of native plant species. Plant Cell Tissue Organ Cult..

[59] Ipsen J.H., Mouritsen O.G., Zuckermann M.J. (1989). Theory of thermal anomalies in the specific heat of lipid bilayers containing cholesterol. Biophys. J..

[60] Vist M.R., Davis J.H. (1990). Phase Equilibria of Cholesterol/Dipalmitoylphosphatidylcholine Mixtures: ^2^H Nuclear Magnetic Resonance and Differential Scanning Calorimetry. Biochemistry.

[61] Veatch S.L., Keller S.L. (2003). Separation of liquid phases in giant vesicles of ternary mixtures of phospholipids and cholesterol. Biophys. J..

[62] Beck J.G., Mathieu D., Loudet C., Buchoux S., Dufourc E.J. (2007). Plant sterols in “rafts”: A better way to regulate membrane thermal shocks. FASEB J..

[63] Bartels T., Lankalapalli R.S., Bittman R., Beyer K., Brown M.F. (2008). Raftlike mixtures of sphingomyelin and cholesterol investigated by solid-state ^2^H NMR spectroscopy. J. Am. Chem. Soc..

[64] Grosjean K., Mongrand S., Beney L., Simon-Plas F., Gerbeau-Pissot P. (2015). Differential Effect of Plant Lipids on Membrane Organization. J. Biol. Chem..

[65] Sha R., Meng Q. (2016). Antifungal activity of rhamnolipids against dimorphic fungi. J. Gen. Appl. Microbiol..

[66] Avis T.J., Bélanger R.R. (2001). Specificity and mode of action of the antifungal fatty acid cis-9-heptadecenoic acid produced by *Pseudozyma flocculosa*. Appl. Environ. Microbiol..

[67] Wise C., Falardeau J., Hagberg I., Avis T.J. (2014). Cellular Lipid Composition Affects Sensitivity of Plant Pathogens to Fengycin, an Antifungal Compound Produced by *Bacillus subtilis* Strain CU12. Phytopathology.

[68] Rella A., Farnoud A.M., Del Poeta M. (2016). Plasma membrane lipids and their role in fungal virulence. Prog. Lipid Res..

[69] Gronnier J., Gerbeau-Pissot P., Germain V., Mongrand S., Simon-Plas F. (2018). Divide and Rule: Plant Plasma Membrane Organization. Trends Plant Sci..

[70] Morikawa T., Mizutani M., Aoki N., Watanabe B., Saga H., Saito S., Oikawa A., Suzuki H., Sakurai N., Shibata D. (2006). Cytochrome P450 CYP710A Encodes the Sterol C-22 Desaturase in Arabidopsis and Tomato. Plant Cell.

[71] Kazan K., Gardiner D.M. (2017). Targeting pathogen sterols: Defence and counterdefence?. PLoS Pathog..

[72] Gamir J., Darwiche R., Van’t Hof P., Choudhary V., Stumpe M., Schneiter R., Mauch F. (2017). The sterol-binding activity of PATHOGENESIS-RELATED PROTEIN 1 reveals the mode of action of an antimicrobial protein. Plant J..

[73] Mas A., Navarro-Pedreño J., Cooke D.T., James C.S. (1994). Characterization and lipid composition of the plasma membrane in grape leaves. Phytochemistry.

[74] Borner G.H.H., Sherrier D.J., Weimar T., Michaelson L.V., Hawkins N.D., Macaskill A., Napier J.A., Beale M.H., Lilley K.S., Dupree P. (2005). Analysis of Detergent-Resistant Membranes in Arabidopsis. Evidence for Plasma Membrane Lipid Rafts. Plant Physiol..

[75] Laloi M., Perret A.-M., Chatre L., Melser S., Cantrel C., Vaultier M.-N., Zachowski A., Bathany K., Schmitter J.-M., Vallet M. (2007). Insights into the role of specific lipids in the formation and delivery of lipid microdomains to the plasma membrane of plant cells. Plant Physiol..

[76] Chalbi N., Martínez-Ballesta M.C., Youssef N.B., Carvajal M. (2015). Intrinsic stability of Brassicaceae plasma membrane in relation to changes in proteins and lipids as a response to salinity. J. Plant Physiol..

[77] Keinath N.F., Kierszniowska S., Lorek J., Bourdais G., Kessler S.A., Shimosato-Asano H., Grossniklaus U., Schulze W.X., Robatzek S., Panstruga R. (2010). PAMP (pathogen-associated molecular pattern)-induced changes in plasma membrane compartmentalization reveal novel components of plant immunity. J. Biol. Chem..

[78] Hao H., Fan L., Chen T., Li R., Li X., He Q., Botella M.A., Lin J. (2014). Clathrin and Membrane Microdomains Cooperatively Regulate RbohD Dynamics and Activity in Arabidopsis. Plant Cell.

[79] Berendsen H.J.C., Van Der Spoel D., Van Drunen R. (1995). GROMACS: A message-passing parallel molecular dynamics implementation. Comput. Phys. Commun..

[80] Abraham M.J., Murtola T., Schulz R., Páll S., Smith J.C., Hess B., Lindahl E. (2015). GROMACS: High performance molecular simulations through multi-level parallelism from laptops to supercomputers. Softw. X.

[81] Malde A.K., Zuo L., Breeze M., Stroet M., Poger D., Nair P.C., Oostenbrink C., Mark A.E. (2011). An Automated Force Field Topology Builder (ATB) and Repository: Version 1.0. J. Chem. Theory Comput..

[82] ATB|Topology Converter. https://atb.uq.edu.au/index.py?tab=topology_converter.

[83] Jämbeck J.P.M., Lyubartsev A.P. (2012). An extension and further validation of an all-atomistic force field for biological membranes. J. Chem. Theory Comput..

[84] Jämbeck J.P.M., Lyubartsev A.P. (2012). Derivation and systematic validation of a refined all-atom force field for phosphatidylcholine lipids. J. Phys. Chem. B.

[85] Kirschner K.N., Yongye A.B., Tschampel S.M., González-Outeiriño J., Daniels C.R., Foley B.L., Woods R.J. (2008). GLYCAM06: A generalizable biomolecular force field. Carbohydrates. J. Comput. Chem..

[86] Berendsen H.J.C., Postma J.P.M., van Gunsteren W.F., Hermans J. (1981). Interaction Models for Water in Relation to Protein Hydration. Intermolecular Forces.

[87] Essmann U., Perera L., Berkowitz M.L., Darden T., Lee H., Pedersen L.G. (1995). A smooth particle mesh Ewald method. J. Chem. Phys..

[88] Bussi G., Donadio D., Parrinello M. (2007). Canonical sampling through velocity rescaling. J. Chem. Phys..

[89] Hess B., Bekker H., Berendsen H.J.C., Fraaije J.G.E.M. (1997). LINCS: A Linear Constraint Solver for Molecular Simulations. J. Comput. Chem..

[90] Parrinello M., Rahman A. (1981). Polymorphic transitions in single crystals: A new molecular dynamics method. J. Appl. Phys..

[91] Nosé S., Klein M.L. (1983). Constant pressure molecular dynamics for molecular systems. Mol. Phys..

[92] Michaud-Agrawal N., Denning E.J., Woolf T.B., Beckstein O. (2011). MDAnalysis: A toolkit for the analysis of molecular dynamics simulations. J. Comput. Chem..

[93] Gowers R.J., Linke M., Barnoud J., Reddy T.J.E., Melo M.N., Seyler S.L., Domański J., Dotson D.L., Buchoux S., Kenney I.M. MDAnalysis: A Python Package for the Rapid Analysis of Molecular Dynamics Simulations. Proceedings of the 15th Python in Science Conference.

[94] Visual Molecular Dynamic. https://www.ks.uiuc.edu/Research/vmd/.

[95] Humphrey W., Dalke A., Schulten K. (1996). VMD: Visual molecular dynamics. J. Mol. Graph..

[96] Hoffman R.E., Becker E.D. (2005). Temperature dependence of the ^1^H chemical shift of tetramethylsilane in chloroform, methanol, and dimethylsulfoxide. J. Magn. Reson..

[97] Davis J.H., Jeffrey K.R., Bloom M., Valic M.I., Higgs T.P. (1976). Quadrupolar echo deuteron magnetic resonance spectroscopy in ordered hydrocarbon chains. Chem. Phys. Lett..

[98] Buchoux S. Nmrdepaker in Launchpad. https://launchpad.net/nmrdepaker.

[99] Burnett L.J., Muller B.H. (1971). Deuteron Quadrupole Coupling Constants in Three Solid Deuterated Paraffin Hydrocarbons: C_2_D_6_, C_4_D_10_, C_6_D_14_. J. Chem. Phys..

