# Supplementary figures and images for: Exploring the Dual Interaction of Natural Rhamnolipids with Plant and Fungal Biomimetic Plasma Membranes through Biophysical Studies
Source: Int J Mol Sci. 2019 Feb 26;20(5):1009. doi: 10.3390/ijms20051009 (PMC6429473; doi:10.3390/ijms20051009)

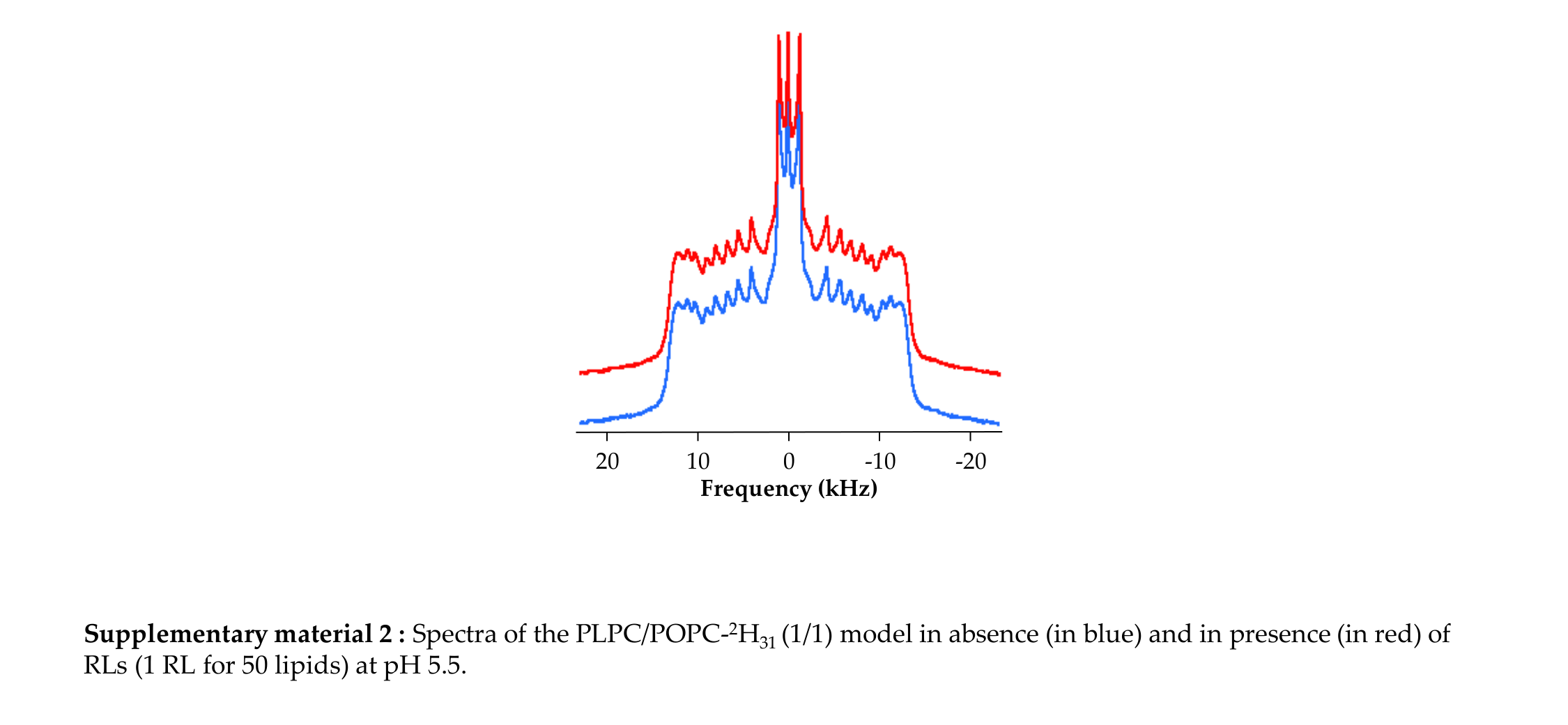

Supplement: Supplementary file 1 [file ijms-20-01009-s001.zip › Supplemental/Supplemental2-Figure S1.tiff]

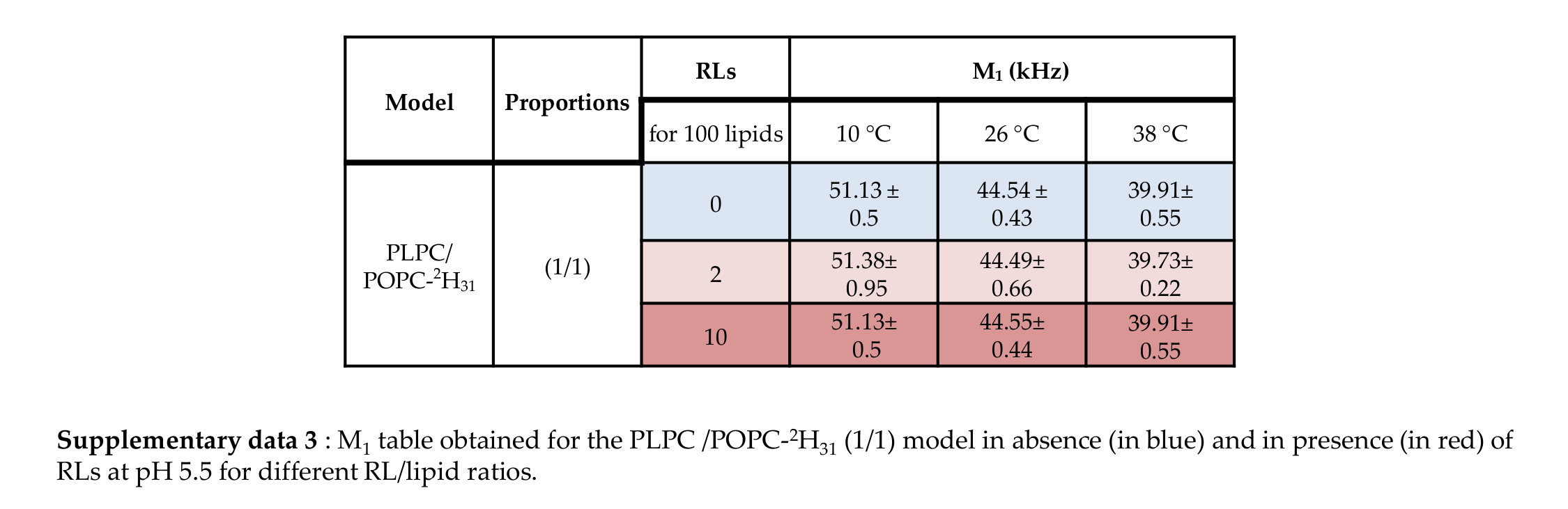

Supplement: Supplementary file 1 [file ijms-20-01009-s001.zip › Supplemental/Supplemental3-Table S1.tiff]

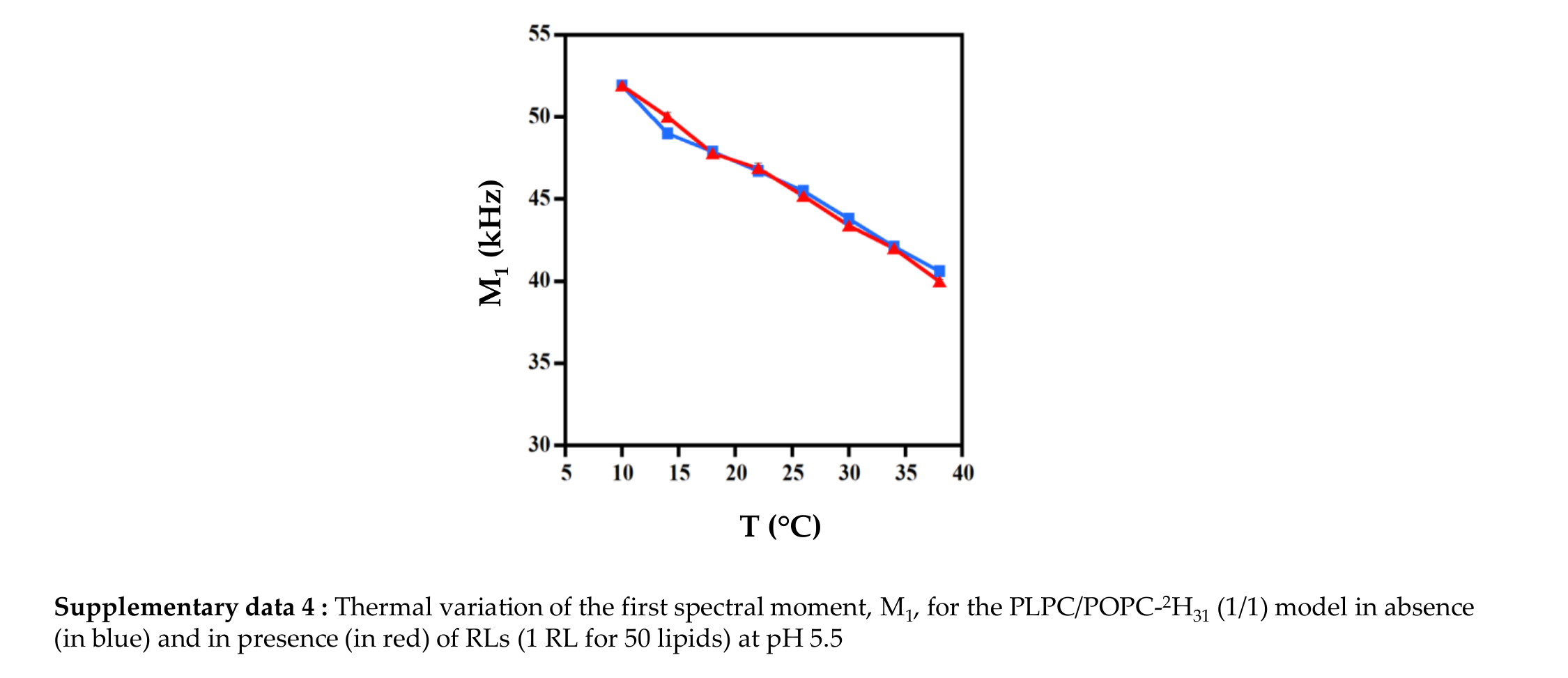

Supplement: Supplementary file 1 [file ijms-20-01009-s001.zip › Supplemental/Supplemental4-Figure S2.tiff]

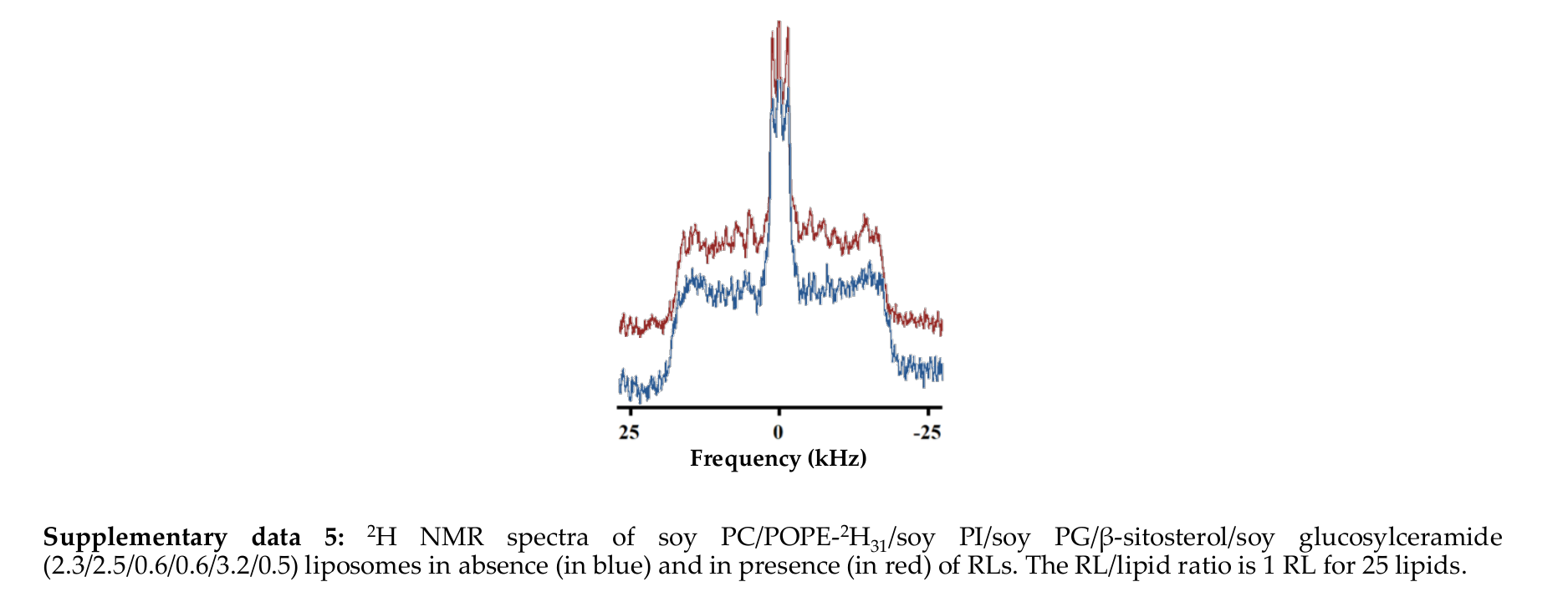

Supplement: Supplementary file 1 [file ijms-20-01009-s001.zip › Supplemental/Supplemental5-Review-Figure S3.tiff]
